# Comparison of the epidemiology and co-morbidities of heart failure in the pediatric and adult populations: a retrospective, cross-sectional study

**DOI:** 10.1186/1471-2261-6-23

**Published:** 2006-05-25

**Authors:** Gregory Webster, Jie Zhang, David Rosenthal

**Affiliations:** 1 Department of Pediatrics, University of California, San Francisco, CA, USA; 2 Department of Statistics, Stanford University, Stanford, CA, USA; 3 Department of Pediatrics, Stanford University, Stanford, CA, USA

## Abstract

**Background:**

Heart failure is a clinical syndrome that is associated with a significant number of interventional procedures and has received a large amount of scrutiny in the adult literature; however, the epidemiology in children is less well described.

**Methods:**

We analyzed two large, commercially available inpatient datasets collected in 1997 by the Agency for Healthcare Research and Quality: the Kids' Inpatient Database and the National Inpatient Study, accounting for 50% of the U.S. pediatric discharges and 20% of the U.S. adult discharges in 1997.

**Results:**

The database contained 5,610 children and 732,752 adults with a diagnosis of HF. When compared with the adult sample, the pediatric sample showed a higher proportion with cardiac procedures (61.4% vs. 0.28%, p < 0.01), a higher prevalence of congenital heart disease (61% versus 0.3%, p < 0.01), a higher percentage of male patients (50% pediatric vs. 44% adult, p < 0.01), and a lower percentage of white patients (40.9% vs. 65.6%, p < 0.01). Children had a significantly different spectrum of co-morbidities compared with adults. There was no difference in mortality rate between children and adults (7.5% vs. 7.9%, p = NS).

**Conclusion:**

There are significant differences in the epidemiological profile of children and adults with heart failure. Children suffer from different types of co-morbidities and require different procedures in the hospital setting. As such, children with heart failure who are hospitalized may require significantly different facilities, management and therapeutic intervention than adults with similar symptoms.

## Background

Heart failure (HF) is a well-recognized clinical syndrome that affects both children and adults in the United States. HF in adults has received considerable attention, with multiple large, randomized trials that have evaluated the etiology and therapy of this condition. For example, the Framingham Heart Study has tracked data on adult cardiac disease since 1948 and numerous more recent trials have evaluated specific therapies for systolic dysfunction [1-12]. However, the pediatric population is more difficult to study. It is difficult to enroll large numbers of children in prospective studies, consent is more challenging to obtain and the definition of suitable endpoints remains challenging.

Previous studies in children such as the 1985 Baltimore-Washington Infant Study have described the incidence of congenital heart disease, but have not focused on heart failure [13]. Recently the Prospective Pediatric Cardiomyopathy Registry reported on the incidence of pediatric cardiomyopathy (but not HF) in 2 regions of the United States, suggesting an incidence of 1.13 cases per 100,000 children [14]. A population-based study conducted in Finland over a longer time frame (11 years) had similar findings [15]. However, heart failure due to other therapies (chemotherapy-induced damage, or heart failure due to congenital heart disease) was specifically excluded and likely comprises a major component of pediatric HF [16]. Other studies of pediatric HF or cardiomyopathy have had a limited sample size or have been from limited geographic regions [17-19].

The annual incidence of dilated cardiomyopathy in adults is 2 to 8 cases per 100,000 in the US and Europe. The prevalence in adults in 1992 was estimated at 26 individuals per 100,000 members of the population [20].

We are not aware of any comparisons between the characteristics of adults with HF and children with HF. There are no reports of complicating co-morbidities in these two populations and the underlying prevalence of HF in these populations has not been compared. Given the importance of understanding the differences between these populations, we have examined a national database compiled over one calendar year containing both pediatric and adult admissions. From this database, we have taken all known cases of heart failure and detailed the co-morbidities associated with the diagnosis, the types of procedures that those patients receive, the length that they stay in the hospital setting and the outcome of their hospital stays. In doing so, we have highlighted some of the differences between the adult and pediatric population with heart failure.

## Methods

### Design and setting

We used two datasets in this study: the Kids' Inpatient Database (KID) and the National Inpatient Study (NIS). Both are commercially available datasets compiled by the Agency for Healthcare Research and Quality (AHRQ) as part of the Healthcare Utilization Project (HCUP). Both databases are subsets of the same larger project. The data were gathered and coded with identical methods. The KID is a pediatric database that is culled from a larger dataset that represents those admissions from 0 to 18 years of age. The KID and NIS are nationwide databases that contain inpatient stay information collected from hospital discharge records. Both databases cover the calendar year 1997. These records are maintained by hospitals, state agencies, and other private data organizations. These data were collected from all discharges by each hospital, compiled by state data organizations and edited and checked by AHRQ. Full documentation for the NIS and KID databases is available through HCUP [21,22].

### Patients and hospitals

The 1997 KID contains approximately 1.9 million pediatric discharges, representing approximately 50% of the pediatric discharges in the United States for the year 1997. It includes data from 22 states. The 1997 NIS contains approximately 7.1 million discharges of all ages from over 1,000 hospitals, containing approximately 20% of adult discharges in the United States for the year 1997.

In both datasets, the included hospitals range from major teaching hospitals to community hospitals. Both datasets include information on all patients, regardless of payer, including those covered by Medicaid, private insurance and the uninsured. Data is grouped into over 100 categories, covering both clinical and non-clinical information. All data files contained a principal diagnosis as coded by the physician or staff member at discharge as well as up to 14 additional diagnoses for each case. All data files contained up to 15 procedure codes, identified by ICD-9-CM code.

### Data collection

Diagnoses consistent with heart failure were identified using the International Classification of Disease code (ICD-9-CM). We included ICD-9 codes 398.91 (rheumatic heart failure, congestive); 402.01, 402.11, and 402.91 (hypertensive heart disease with congestive heart failure); 404.01, 404.11, 404.91, 404.03, 404.13, and 404.93 (hypertensive heart and renal disease with congestive heart failure); 428.xx (congestive heart failure); and 429.4 (cardiac failure following surgery, excluding immediate postoperative failure [997.1]). Each diagnostic code was converted to a diagnostic category based on the Clinical Classification Software (CCS) available from the HCUP website. After the initial selection by ICD-9 code, all subsequent analysis was based on these CCS codings. Relevant procedures were identified using the CCS. Full documentation on CCS has been described by HCUP previously [23].

### Analysis

The population was divided into three groups, those under one year of age (infants), those between one and 18 years of age (children), and those over 18 years of age (adults). These criteria were chosen prior to analysis based on the clinical observation that the etiology of heart failure differs before and after one year of age [14]. Where the two pediatric groups were not significantly different, the results were pooled to simplify presentation of the data. Cases were analyzed for the number of co-morbid diagnoses in each group. Co-morbid diagnoses were divided into system-based categories and all CCS diagnostic codes were assigned to a system-based category by the authors.

The CCS procedure codes were similarly divided by the authors into system-based categories with a large "miscellaneous" category that contained both non-classifiable procedures and those procedures that were classifiable into multiple system-based categories.

Chi square analysis was utilized to compare the adult and pediatric populations in all categorical variables. Sub-testing was done using the Yates correction and the Bonferroni inequality was used to allow for the fact that multiple comparisons were being done. For continuous variables, ANOVA testing was used.

## Results and discussion

We identified 5,610 people 18 years old or less and 732,752 people greater than 18 years old with heart failure. In our sample population, 3,176 children were younger than one year of age (infants). HF was the primary diagnosis in 564 infants (18%) and was a secondary diagnosis in 2,612 infants (82%). There were 2,434 patients between one year and 18 years of age (children). HF was the primary diagnosis in 614 children (25%) and was a secondary diagnosis in the remaining 1,820 children (75%).

The data on gender are summarized in Table 1. Unlike in older patients, where there is a substantial over-representation of female patients, we find that children have a nearly identical proportion of male and female patients.

**Table 1:** Gender Comparison

	Gender (%)	
Age Group	Male	Female
Age 0 – 18	2792 (49.8)	2817 (50.2)
Age > 18	323972 (44.2)	408737 (55.8)

p-value	<0.01	<0.01

The pediatric sample (ages 0–18) had a significantly greater percentage of non-white patients when compared to their adult counterparts (Table 2). However, in both pediatric and adult groups, whites make up the greatest number overall, followed by blacks and then Hispanics. Note that the numbers do not sum to 100% since not all patients in the database were assigned a code for race at the time of discharge.

**Table 2:** Racial Demographics

				Races comprising Non-white Category			
Age Group	White	Non-white	Black	Hispanic	Asian/Pacific Islander	Native American	Other
Age 0 – 18	2295 (40.9)	1970 (35.1)	797 (14.2)	757 (13.5)	178 (3.2)	28 (0.5)	210 (3.7)
Age > 18	480867 (65.6)	111624 (15.2)	69520 (9.5)	29980 (4.1)	5488 (0.7)	766 (0.1)	5870 (0.8)

p-value	< 0.01	< 0.01					

Note: numbers in parenthesis represent % of total population in each age range

Pediatric hospital length of stay was appreciably longer than adults, with infants having the longest stays (Table 3). The presence or absence of heart failure as a primary diagnosis did not reliably predict length of stay across the three groups.

**Table 3:** Age and Length of Stay

Age (years)	Length of Stay (days)
0 – 1	15.52*
≥1 and ≤18	11.1*
> 18	7.43*

* All comparisons p < 0.01 versus the other two comparison groups.

Many patients were also documented as having important co-morbidities. As would be expected, the incidence of congenital cardiac disease was greater in both infants and children when compared with the adult population. However, there was a striking decrease in the incidence of congenital cardiac disease in children when compared with infants, which may be due to the likelihood of having congenital defects repaired in infancy, reducing the likelihood of hospitalization due to left to right shunting lesions. Infants and children have a high incidence of non-cardiac congenital lesions. The other identified diagnostic categories are summarized in Table 4.

**Table 4:** Subspecialty Comorbid Diagnoses

	Percent of patients with diagnosis				
Diagnosis	Age < 1 yr	Age ≥ 1 yr and ≤ 18 yr	Age >18 yr	Infants vs. Children p-value*	Pediatric vs. Adult p-value^+^
Congenital, Cardiac	82.27%	34.26%	0.28%	<0.01	<0.01
Congenital, Non-cardiac	30.64%	14.46%	0.36%	<0.01	<0.01
Neurological	8.25%	21.2%	34.13%	<0.01	<0.01
Hematological/Oncologic	14.29%	25.84%	31.38%	<0.01	<0.01
Infectious Diseases	33.56%	36.11%	47.64%	0.05	<0.01
Pulmonary	30.2%	35.09%	22.87%	<0.01	<0.01
Rheumatology	0.38%	2.44%	7.13%	<0.01	<0.01
Ophthalmology	2.15%	3.23%	5.53%	<0.01	<0.01
Orthopedics	1.75%	6.53%	17.13%	<0.01	<0.01
Otolaryngology	3.83%	4.73%	2.02%	0.14	<0.01
Obstetrics and Gynecology	0.10%	2.59%	1.16%	<0.01	<0.01
Renal	8.45%	18.93%	30.88%	<0.01	<0.01
Endocrine	19.08%	20.05%	26.73%	<0.01	<0.01
Gastroenterological	3.83%	4.72%	2.02%	0.37	<0.01

* Infants vs. children comparison is between infants (age <1 yr) and children (ages ≥ 1 year and ≤ 18 years)

+ Pediatric vs. adult comparison is between all infants and children (age > 0 and ≤ 18 years) and all adults (age > 18 years)

The frequency of cardiac procedures is shown in Table 5. Pediatric patients have a higher proportion of cardiac bypass procedures when compared with adult patients. This underscores the high prevalence of surgical management for serious structural problems in pediatrics. The rates of intubation are significantly higher in pediatrics than in adults, which probably reflects the increased prevalence of surgical management in pediatrics. In addition, cardiac transplantation, while infrequent in all age groups, was significantly more common in children than in adults.

**Table 5:** Procedural Comparison

	Percent of patients undergoing procedure			
	Age < 1 yr	Age ≥ 1 yr and ≤ 18 yr	Age >18 yr	p-value*
All Procedures	82.2%	34.2%	0.3%	<0.01
- Intubation	28.2%	14.9%	6.9%	<0.01
- Cardiac Bypass	25.9%	16.2%	2.6%	<0.01
- Cardioversion	2.2%	2.6%	1.8%	0.01
- Cardiac Transplantation	0.5%	3.3%	0.1%	<0.01
- Other Cardiac Procedure	82.3%	34.3%	0.3%	<0.01

* P-value is due to variability between pediatric patients (age 0 – 18 years) and adults (age > 18 years).

Not surprisingly, stays that required the use of cardioversion were associated with increased risk of mortality in all age groups. Findings for intubation were similar. These data are shown in Table 6.

**Table 6:** Mortality Comparison by Procedure

	Mortality (%)			Mortality (%)		
				
	With Cardioversion	Without Cardioversion	p-value	With Intubation	Without Intubation	p-value
Age < 1 yr.	47.1	6.3	<0.01	15.8	3.8	<0.01
Age ≥ 1 and ≤ 18 yrs.	48.4	6.7	<0.01	23.8	5.0	<0.01
Age > 18 yrs.	36.5	6.8	<0.01	36.3	5.8	<0.01

There was no difference in overall mortality between children and adults (7.5% vs. 7.9%, p = NS), which is probably a serendipitous finding given the significant differences in etiologies, co-morbidities, and treatment approaches between the two groups.

We describe here differences between pediatric and adult heart failure patients in the hospital setting. It is challenging to gather pediatric data that can be directly compared against adult data since almost all large trials have been exclusively in the adult population. We have explored a single database, designed for financial data gathering; and, from there, we have extracted relevant clinical information to compare the two populations. Since it is only in the last several years that data of this type have been made available, this has rarely been done previously and never in HF to our knowledge.

Clear differences exist between pediatric and adult HF patients. The overall difference between the numbers of procedures that children undergo versus adults is striking. A likely cause for this difference is the discrepancy between the frequencies of congenital heart disease in the two populations. Our study supports the observation that congenital heart disease is a common causative factor in pediatric heart failure, while it is a much less common reason for HF in adults. As the number of adults with repaired congenital heart disease increases, the number of cases of HF in adults due to congenital heart disease will also increase, but our data suggest that – despite this anticipated growth – congenital heart disease is not likely to become a common cause of HF in adults.

In addition, the prevalence of other congenital anomalies (including non-cardiac) is higher in hospitalized children than in hospitalized adults. There are three reasons that may account for this observation. First, patients with other congenital disorders may have excess childhood mortality and would therefore not be included in adult samples. In addition, there may be a recording bias toward classifying problems as congenital by the physicians who care for children. Finally, coronary artery disease is a major cause of HF and subsequent hospitalization in adults but it is rare in children, and this etiology is presumably not related to the presence of congenital anomalies.

Our study suggests that there are other significant differences between children and adults with HF. Adults are significantly more likely to have neurological, hematological and oncologic, infectious disease, renal and endocrine abnormalities, suggesting that adults are often sick with multi-system disease that may not be directly linked to the etiology of their heart failure. However, the association between HF and other congenital diseases in children suggests that children may be more likely to have syndromic conditions that influence their outcome. Further study is necessary to determine what percentage of children and adults have significant mortality due to those co-morbidities.

While in the hospital, children were significantly more likely than adults to undergo intubation, cardiac bypass, cardioversion and other cardiac procedures. Hospital stays were significantly longer in the pediatric population, perhaps due to the surgical management of major structural anomalies. In contrast, adults with HF experienced a low utilization of intubation, cardiac bypass and other cardiac procedures, suggesting that the major components of their regimen were medical, as opposed to surgical, therapies.

A major limitation of our study lies in the fact that we had access only to a large, commercially available dataset. Therefore, it was impossible to correlate specific diagnoses with chart review since we had no access to individual patient documentation. Similarly, since the dataset was primarily designed for utilization analysis, we were unable to further subdivide the operative procedures into subcategories that would allow us to describe specific interventions. However, the benefit of using a large, if categorically limited, dataset is that it allows us a well-powered study. This study indicates that the significant differences in interventions in patients with heart failure suggest that the etiology, and therefore subsequent care, of children's heart failure differ significantly between children and adults.

## Conclusion

We have found that significantly different interventions are utilized for children and adults in the hospital setting. As such, children with heart failure who are hospitalized may require significantly different facilities, management and therapeutic intervention than adults with similar symptoms. Given the high rate of procedural interventions for children with congestive heart failure, close collaboration between cardiologists and cardiothoracic surgeons is imperative to optimize care for these children.

## Competing interests

The author(s) declare that they have no competing interests.

## Authors' contributions

GW participated in the design and coordination of the study, participated in the statistical analysis and drafted the manuscript. JZ was responsible for the primary statistical analysis. DR conceived of the study concept, participated in the design, reviewed the statistical analysis and edited the manuscript. All authors read and approved the final manuscript. GW received support for this work from the Stanford University Medical Scholars Award for Biomedical Research.

## Pre-publication history

The pre-publication history for this paper can be accessed here:


